# Identification of differentially expressed genes implicated in peel color (red and green) of *Dimocarpusconfinis*

**DOI:** 10.1186/s40064-016-2743-y

**Published:** 2016-07-15

**Authors:** Fan Jiang, Xiu-ping Chen, Wen-shun Hu, Shao-quan Zheng

**Affiliations:** Fujian Fruit Breeding Engineering Technology Research Center for Longan and Loquat, Fuzhou, 350013 Fujian China; Fruit Research Institute, Fujian Academy of Agricultural Science, Fuzhou, 350013 Fujian China

**Keywords:** *Dimocarpus confinis*, Peel color, Anthocyanins (ACs), Sequence analysis, Differentially expressed genes (DEGs)

## Abstract

Nowadays, there are few reports about regulatory genes implicated in peel color of longan. The basic genetic research of longan has been in stagnation for a long time as a lack of transcriptomic and genetic information. To predict candidate genes associated with peel color, Gene Functional Annotation and Coding Sequence prediction were used to perform functional annotation for our assembled unigenes and investigate differentially expressed genes (DEGs) of fruitlet peels from Longli (*Dimocarpus confinis*). Finally, a total of 24,044 (44.19 %) unigenes were annotated at least in one database after BLAST search to NCBI non-redundant protein sequence, NCBI non-redundant nucleotide sequences, Kyoto Encyclopedia of Genes and Genomes (KEGG) Ortholog, manually annotated and reviewed protein sequence database (Swiss-Prot), Protein family, Gene Ontology, euKaryotic Ortholog Groups databases. After searching against the KEGG-GENE protein database, a result of 6228 (11.45 %) unigenes were assigned to 245 KEGG pathways. Via comparing the distributions of expression value of all corresponding unigenes from red peel and green peel fruit, it could be intuitively concluded that high similarity was existed in the two distributions; however, on the whole, between two distributions of log RPKM expression value, some differences indicated that expression level in green-peel fruit group is slightly higher than values in red-peel fruit group. Finally, a total of 1349 unigenes were identified as DEGs after blasting the DEGs to public sequence databases, and 32 peel-color-related genes were identified in longan. Our results suggest that a number of unigenes involved in longan metabolic process, including anthocyanin biosynthesis. In addition, *DRF*, *F3H*, *ANS*, *CYP75A1* and *C1* may be the key ones. The study on key genes related to peel color will be contributed to revealing the molecular mechanisms of regulating peel color in woody plants.

**Electronic supplementary material:**

The online version of this article (doi:10.1186/s40064-016-2743-y) contains supplementary material, which is available to authorized users.

## Background

The tropical/subtropical fruit tree longan (*Dimocarpus longan* Lour.) is in the family Sapindaceae that is cultivated all over the world, especially in China, Thailand, Vietnam (Jiang et al. 2002). Nowadays, with the rapid development of the agricultural economy, planting area and field in China has been the largest and highest in the world so far (Wu 2010).

Peel, the pulp section, differentiated and developed from ovary wall. Mature pericarp is generally divided into exocarp, mesocarp, endocarp. As reported, most peels are likely to have a certain characteristics, such as medical use, value-added ingredients for various food applications, anti-mosquito and deodorant (Abdul Aziz et al. 2012; Denis et al. 2013; Rawson et al. 2014). Additionally, another trait of peel is about color, which is one of the main factors that determines consumer preference and market price. Peel color of many other fruits except longan may be varied with the environmental or internal factors changed (González-Talice et al. 2013; Liu et al. 2015; Zhao et al. 2011). In the aspect of external factors, peach peel color changed when processed by bagging with a widely applied Yellow-Paper (Liu et al. 2015), and the intensity of light also plays an fundamental role in color development of apple peel (Zhao et al. 2011). As for the internal factors, pigments, total phenolic and total flavonoid concentration are important factors determining color and internal apple quality (González-Talice et al. 2013), and more and more researches showed anthocyanins (ACs) played an important role in peel color (Liu et al. 2015; Rahim et al. 2014; Wang et al. 2015; Zhao et al. 2013). ACs, a naturally water-soluble pigment of flavonoid family generated from secondary metabolites, are widely distributed in fruits and vegetables, as well, its potential health benefits to humankind provoking an increasing interest in these compounds (Boyer and Hai Liu 2004; Hyson 2011; Stover and Mercure 2007). Anthocyanin plays a photoprotective role in plants under high light or photoinhibition conditions (Close and Beadle 2005; Hoch et al. 2003; Hughes et al. 2005, 2007, 2012; Li et al. 2008; Manetas et al. 2002; Williams et al. 2003). In pear, the higher photoprotective capacity in the sun-exposed peel of red “Anjou” pear than green “Anjou” is mainly attributed to its higher anthocyanin concentration (Li et al. 2008). But, do anthocyanin act on red peel (RP) and green peel (GP) of longan, except for photoprotective of some other fruits? And which of genes involved in anthocyanin biosynthetic pathways play a major role in peel coloration? The structural genes, encoding corresponding enzymes in the anthocyanin biosynthetic pathway, have been cloned from varieties of plants, and several regulatory genes implicated in the activation of coloration have recently been cloned in previous studies as well (Espley et al. 2007; Goff et al. 1992; Goodrich et al. 1992; Niu et al. 2010; Schwinn et al. 2006). In apple, there were two cultivars with red and green peel, anthocyanins and flavonols elevated when turning shaded peel (shaded peel of the two cultivars were green) to sun exposure for a week, along with green peel to red peel. As well, exposure of the shaded peel to full sun caused marked up-regulation of expression levels of *MYB10* (a transcriptional factor in the regulation of anthocyanin biosynthesis) and seven structural genes in anthocyanin synthesis (*PAL*, *CHS*, *CHI*, *F3H*, *DFR1*, *LDOX*, and *UFGT*) (Feng et al. 2013). Besides, myeloblastosis (*MYB*) was also proved to play an important role in regulating peel color in some fruits, such as peach, pear, apple (Feng et al. 2013; Rahim et al. 2014; Sun et al. 2013; Yang et al. 2015), meanwhile, *MYB10.1* and *MYB10.3* have positive correlation with the expression of key structural genes of the anthocyanin pathway in peach, such as chalcone synthase flavanone 3-hydroxylase (*F3H*), and UDP-glycose: flavonoid glycosyltransferase (*UFGT*) (Rahim et al. 2014). *PyMADS18* was reputed to be involved in anthocyanin accumulation and regulation of anthocyanin synthesis in early fruit development of pear (Wu et al. 2013), and anthocyanidin synthase (*ANS*) and UDP-glucose flavonoid 3-O-glucosyltransferase (*UFGT*), whose different expressions led to the coloration differences between occidental and oriental pears, were speculated to be key genes for anthocyanin biosynthesis for red-skinned pear (Yang et al. 2015).

**Fig. 1:**
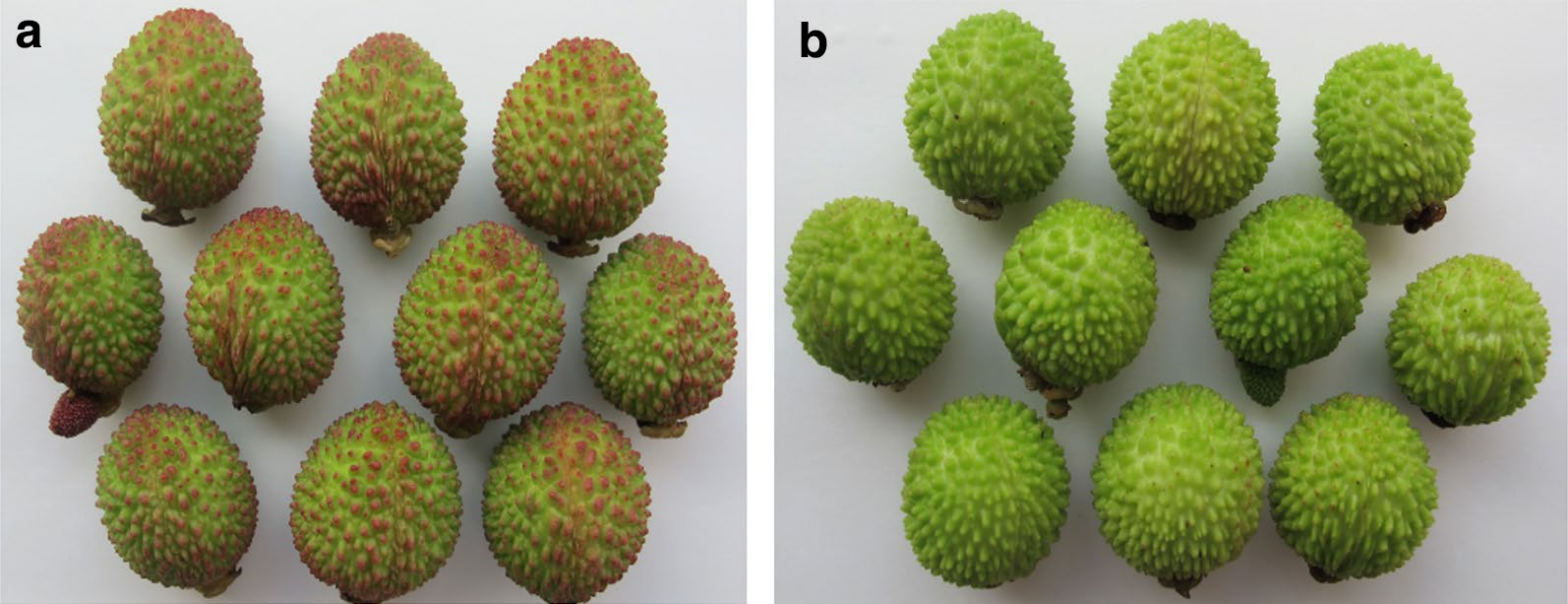
A special longan germplasm resource of Longli. **a** Longli, whose fruitlet peels showed *red* in the fruit development process. **b** Longli, whose fruitlet peels showed *green* in the fruit development process

In our previous study, anthocyanin content and composition in the peel of *Dimocarpus confinis* (Jiang et al. 2014), a relative species of *Dimocarpus Lour.*, were examined using a HPLC method. The results showed that anthocyanin content was 18.60 ± 5.12 mg kg^−1^ (FW) in red peel, and was significantly higher than in light red peel and in blue green peel by 6.8 times and 33.2 times, respectively. In the present study, a special longan germplasm resource of Longli from Fujian Province, whose fruitlet peels showed red and green in the fruit development process individually (Fig. 1a, b), were applied to initially revealing the molecular mechanism of regulating peel color. So in this study, we firstly sequenced the transcriptomes of Red Peel and Green Peel longan using Illumina technology. We focused on the discovery of encoding enzymes involved in the anthocyanin biosynthetic pathway and obtained sets of up-regulated and down-regulated genes from red and green peel of longan, and finally identified some candidate genes related to anthocyanin synthesis in longan peel. The assembled annotated transcriptome sequences provide a valuable genomic resource to further understand the molecular mechanism of regulating peel color.

## Methods

### Plant materials

As a special longan germplasm resource, Longli (Genebank number GPLY0124) was cultured in the National Field Genebank for Longan and Loquat (Fuzhou, Fujian, China). After flowering 30 days, healthy peel tissue from fruitlet period manifested as red and green was collected from the fruit of Longli and immediately frozen in liquid nitrogen, and stored at −80 °C until further processing.

### RNA preparation

The TRIzol^®^ reagent (Invitrogen) was used to extract total RNA from the peels of red and green longli (*D. confinis*) according to the manufacturer’s instructions (Invitrogen, USA). The purity of all RNA samples was assessed by and the RNA quality was tested using a 1 % ethidium bromide-stained agarose gels. RNA integrity was assessed using the RNA Nano 6000 Assay Kit of the Agilent Bioanalyzer 2100 system (Agilent Technologies, CA, USA).

### cDNA synthesis and Illumina sequencing

A total amount of 3 μg RNA, extracted from peels of RP and GP longan, was used as input material for the RNA sample preparations. The samples were treated with RNase-free DNase I (Takara Biotechnology, China). Sequencing libraries were generated using NEBNext^®^Ultra™RNA Library Prep Kit for Illumina^®^ (NEB, USA) following manufacturer’s recommendations and index codes were added to attribute sequences to each sample. Briefly, mRNA was purified from total RNA using poly-T oligo-attached magnetic beads. Fragmentation was carried out using divalent cations under elevated temperature in NEBNext First Strand Synthesis Reaction Buffer (5×). First strand cDNA was synthesized using random hexamer primer and M-MuLV Reverse Transcriptase (RNase H-). Second strand cDNA synthesis was subsequently performed using DNA Polymerase I and RNase H. Remaining overhangs were converted into blunt ends via exonuclease/polymerase activities. After adenylation of 3′ ends of DNA fragments, NEBNext Adaptor with hairpin loop structure were ligated to prepare for hybridization. In order to select cDNA fragments of preferentially 150–200 bp in length, the library fragments were purified with AMPure XP system (Beckman Coulter, Beverly, USA). Then 3 μl USER Enzyme (NEB, USA) was used with size-selected, adaptor-ligated cDNA at 37 °C for 15 min followed by 5 min at 95 °C before PCR. Then PCR was performed with Phusion High-Fidelity DNA polymerase, Universal PCR primers and Index (X) Primer. At last, PCR products were purified (AMPure XP system) and library quality was assessed on the Agilent Bioanalyzer 2100 system.

### Clustering and sequencing

The clustering of the index-coded samples was performed on a cBot Cluster Generation System using TruSeq PE Cluster Kit v3-cBot-HS (Illumia) according to the manufacturer’s instructions. After cluster generation, the library preparations were sequenced on an Illumina Hiseq 2000 platform and paired-end reads were generated.

### Quality control

Raw data (raw reads) of fastq format were firstly processed through in-house perl scripts. In this step, clean data (clean reads) were obtained by removing reads containing adapter, reads containing ploy-N and low quality reads from raw data. At the same time, Q20, Q30, GC-content and sequence duplication level of the clean data were calculated. All the downstream analyses were based on clean data with high quality.

### Transcriptome assembly and annotation

The left files (read1 files) from all libraries/samples were pooled into one big left.fq file, and right files (read2 files) into one big right.fq file. Transcriptome assembly was accomplished based on the left.fq and right.fq using Trinity (Grabherr et al. 2011) with min_kmer_cov set to 2 by default and all other parameters set default. And gene function was annotated based on the following databases: NR (Altschul et al. 1997), NT (Pruitt et al. 2005), PFAM (http://pfam.sanger.ac.uk/) (Finn et al. 2008), KOG/COG (http://www.ncbi.nlm.nih.gov/COG/) (Tatusov et al. 2003), Swiss-Prot (http://www.ebi.ac.uk/uniprot/) (Karp et al. 2001), KO (http://www.genome.jp/kegg/) (Moriya et al. 2007) and GO (http://www.geneontology.org/) (Gotz et al. 2008).

### ESTScan software

ESTScan (http://www.ch.embnet.org/software/ESTScan.html) (Iseli et al. 1999) was performed to detect and extract coding regions from low-quality sequences with high selectivity and sensitivity, which is also able to accurately correct frameshift errors. In the framework of genome sequencing projects, ESTScan could become a very useful tool for gene discovery, for quality control, and for the assembly of consigns representing the coding regions of genes.

### SNP calling

Picard-tools v1.41 and samtools v0.1.18 were used to sort, remove duplicated reads and merge the bam alignment results of each sample. GATK2 software was used to perform SNP calling. Raw vcf files were filtered with GATK standard filter method and other parameters (clusterWindowSize: 10; MQ0 ≥ 4 and [MQ0/(1.0 * DP)] > 0.1; QUAL < 10; QUAL < 30.0 or QD < 5.0 or HRun > 5), and only SNPs with distance >5 were retained.

### Quantification of gene expression levels

Gene expression levels were estimated by RNA-Seq by Expectation Maximization (RSEM) (Li and Dewey 2011) for each sample. RSEM has been regarded as an accurate and user-friendly software tool for quantifying transcript abundances from RNASeq data. By RSEM, clean data were mapped back onto the assembled transcriptome and read-count for each gene was obtained from the mapping results. As RSEM does not rely on the existence of a reference genome, it is particularly useful for quantification with de novo transcriptome assemblies.

### Differential expression analysis

#### For the samples with biological replicates

Differential expression analysis of two conditions/groups was performed using the DESeq R package (1.10.1). DESeq provide statistical routines for determining differential expression in digital gene expression data using a model based on the negative binomial distribution. The resulting p values were adjusted using the Benjamini and Hochberg’s approach for controlling the false discovery rate. Genes with an adjusted p value <0.05 found by DESeq were assigned as differentially expressed.

#### For the samples without biological replicates

Prior to differential gene expression analysis, for each sequenced library, the read counts were adjusted by edgeR program package through one scaling normalized factor.

Differential expression analysis of two samples was performed using the DEGseq (2010) R package. p value was adjusted using q value (Storey and Tibshirani 2003). The q value <0.005 and |log_2_ (FoldChange)| > 1 was set as the threshold for significantly differential expression.

### Enrichment analysis methods

GO enrichment analysis of the differentially expressed genes (DEGs) was implemented by the GOseq R packages based Wallenius non-central hyper-geometric distribution (Young et al. 2010), which can adjust for gene length bias in DEGs. The q value <0.05 was set as the threshold for GO enrichment.

KEGG (Kanehisa et al. 2008) pathway assignments were mapped according to the KEGG database (http://www.genome.jp/kegg/). We used KOBAS (Mao et al. 2005) software to test the statistical enrichment of differential expression genes in KEGG pathways with a q value <0.05 after searching the KEGG protein databases.

## Results

### Gene functional annotation and CDS prediction

In this study, seven different public databases (NR, NT, KO, Swiss-Prot, PFAM, GO and KOG) were used to perform functional annotation for our assembled unigenes (combined red peel fruit and green peel fruit groups), and finally 44.19 % (24,044) of total unigenes were annotated at least in one database, with 30,365 unigenes remaining unannotated in any database. These unannotated unigenes may represent specific transcripts or erroneous assemblies and untranslated regions. For each database, the detailed numbers and percentages of successfully annotated unigenes were shown in the Table 1.

**Table 1:** Overview of number and percentage of annotated unigenes

	Number of unigenes	Percentage (%)
Annotated in NR	21,620	39.73
Annotated in NT	13,117	24.1
Annotated in KO	6353	11.68
Annotated in SwissProt	16,344	30.03
Annotated in PFAM	16,972	31.19
Annotated in GO	18,617	34.21
Annotated in KOG	8675	15.94
Annotated in all databases	2145	3.94
Annotated in at least one database	24,044	44.19
Total unigenes	54,409	100

Furthermore, in order to predict CDS of unigenes, after successively aligning the longan (Red and Green) unigenes to NR, Swiss-Prot and KO databases by using BLASTx, totally 22,550 (41.44 %) unigenes had significant similarity to known protein-coding genes and then the prediction of open reading frames (ORFs) was processed according to the best hit. The remaining 31,859 unigenes with no hits in the above protein databases were scanned again by implementing ESTScan, and the ORFs of 15,785 unigenes were de novo predicted through this method. In total, 38,335 (70 %) unigenes were translated to polypeptide sequence based on homology analysis using BLASTx and ESTScan predictions.

**Fig. 2:**
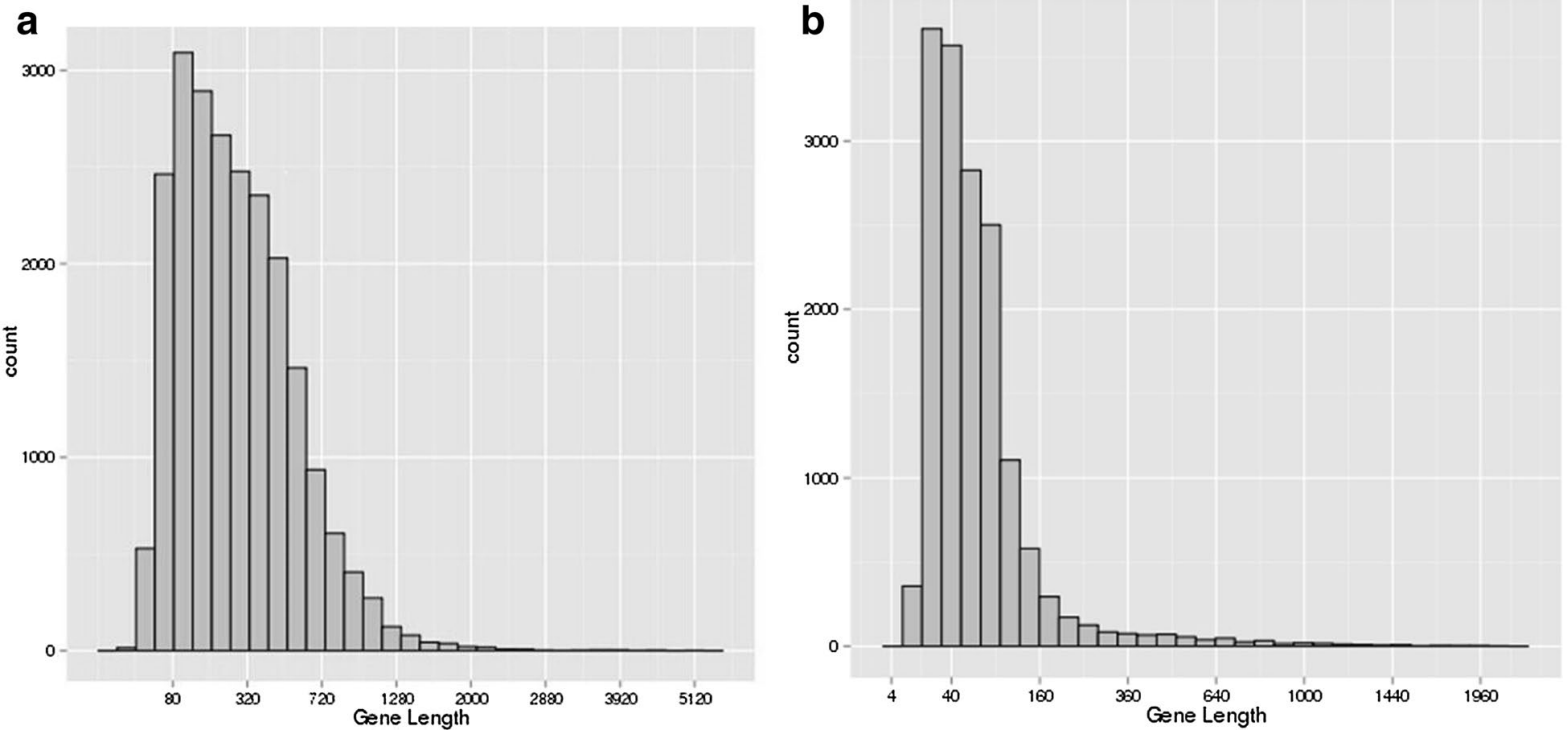
Length distributions of translated proteins predicted by BLAST (**a**) and ESTScan (**b**) respectively

 As shown in Fig. 2, via comparing two length distributions of proteins predicted by BLASTx(A) and ESTScan(B) respectively, majority of these proteins identified from BLASTx are longer than those proteins obtained from ESTScan.

Of above public annotation databases, GO and KOG were used to functionally categorize *Dimocarpus longan* (RP and GP) unigenes.

**Fig. 3:**
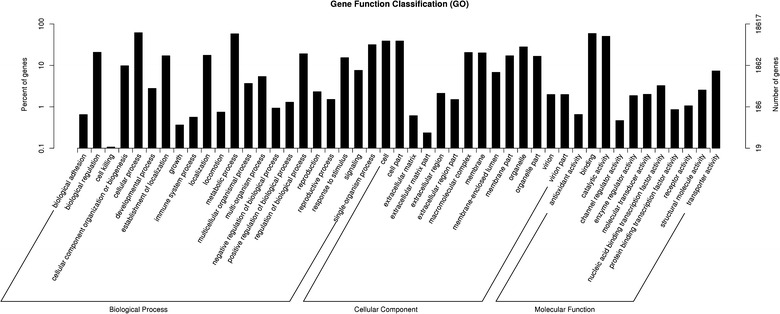
Gene ontology classification of the *D. longan* transcriptome. The unigenes were classified at the second level under three root GO domains: cellular component (CC), molecular function (MF) and biological process (BP). The *right* and *left* *y-axis* indicate the number and the corresponding percentage of a certain ontology within each root domain, respectively. One unigene could be annotated into more than one GO term

As shown in Fig. 3, 18,167 GO-annotated unigenes (34.21 %) were distributed to the three main GO categories (level 1) and 47 sub-categories (level 2). It needs to be additional explained that one unigene could be assigned to more than one GO term.

In biological process (BP) category included 22 sub-categories, we observed a high percentage of unigenes assigned to “cellular process”, “metabolic process”, “single-organism process”, and “biological regulation”. Whereas, the cellular component (CC) category was classified as 14 sub-categories, among which, “cell” was the most enriched category, followed by “cell part”, “organelle” and “macromolecular complex”. With regard to molecular function (MF) category, there were 11 sub-categories involved in “binding”, “catalytic activity” and “transporter activity” (Fig. 3).

**Fig. 4:**
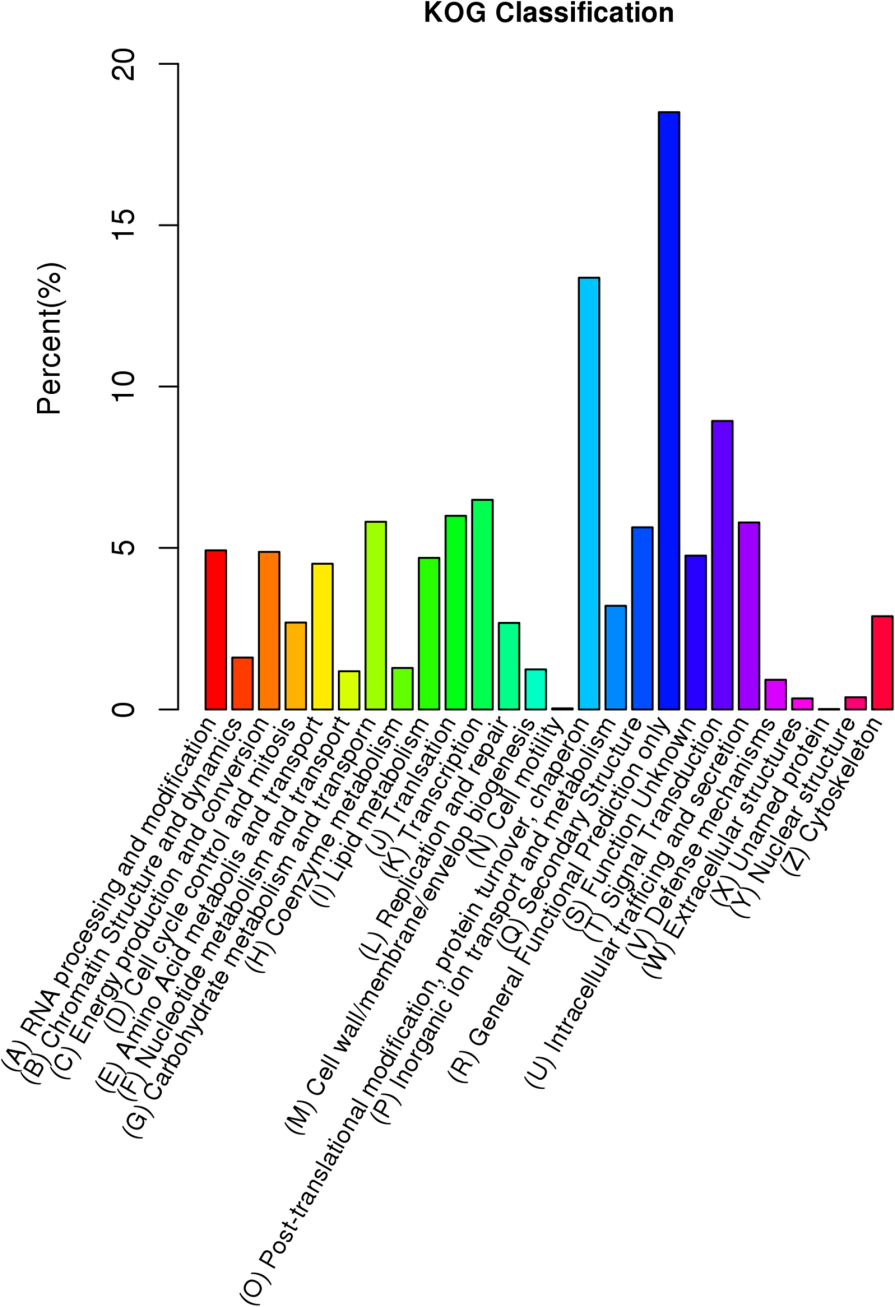
KOG classification of the *D. longan* transcriptome. Each function class was represented by different *capital letters* under the *x-axis*. The *y-axis* denotes the percent of unigenes in a corresponding function class

On the other hand, overall 8675 (15.94 %) unigenes, less than GO results, were annotated based on KOG analysis and assigned to 26 function classes (Fig. 4). Except (R) General Functional Prediction only (1605 unigenes; 18.50 %), the three largest classes were (O) Post-translational modification, protein turnover, chaperon (1160; 13.37 %), (T) Signal Transduction (775; 8.93 %) and (K) Transcription (563; 6.49 %). In addition, 413 (4.76 %) unigenes were assigned to (S) Function Unknown.

The KEGG database, which is commonly used resource for the systematic understanding of the networks of the biological system, can be used to perform pathway-based analysis.

**Fig. 5:**
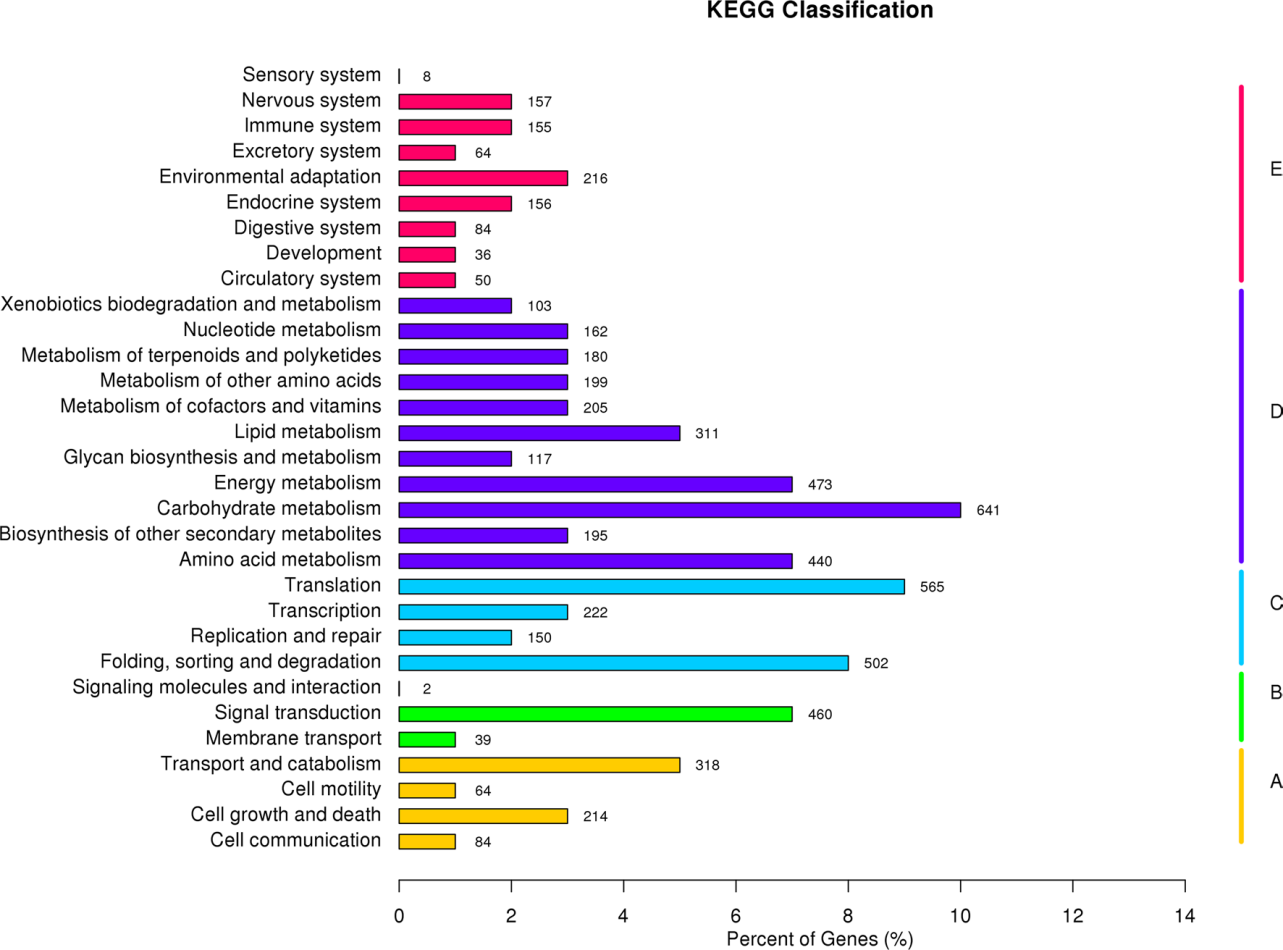
KEGG classification of the *D. longan* transcriptome. Each pathway was represented by different *capital letters* under the *y-axis*. The *x-axis* denotes the percent of unigenes in a corresponding function class. KEGG has five Pathway Hierarchy1: *A*, cellular processes; *B*, environmental information processing; *C*, genetic information processing; *D*, metabolism; *E*, organismal systems

After searching against the KEGG-GENE protein database, as a result, 6228 (11.15 %) unigenes were assigned to 245 KEGG pathways. As shown in Fig. 5, among these pathways, “Carbohydrate metabolism” accounted for the largest proportion (737 unigenes, 11.83 %) followed by “Translation” (610, 9.79 %), “Folding, sorting and degradation” (531, 8.53 %), “Overview” (492, 7.90 %) and “Amino acid metabolism” (451, 7.24 %).

### Analysis of DEGs

To investigate the changes in gene expression and understand the critical genes involved in the trait of peel coloration, clean reads of red- and green-peel fruit groups respectively were mapped (using Bowtie with default parameters) to the transcriptome reference sequences de novo assembled by Trinity and were processed by RSEM. The transcript expression level of each unigene was estimated by Reads Per Kilo bases per Million mapped Reads (RPKM), which provides a useful measure of expression level that accounts for variation in gene length.

**Fig. 6:**
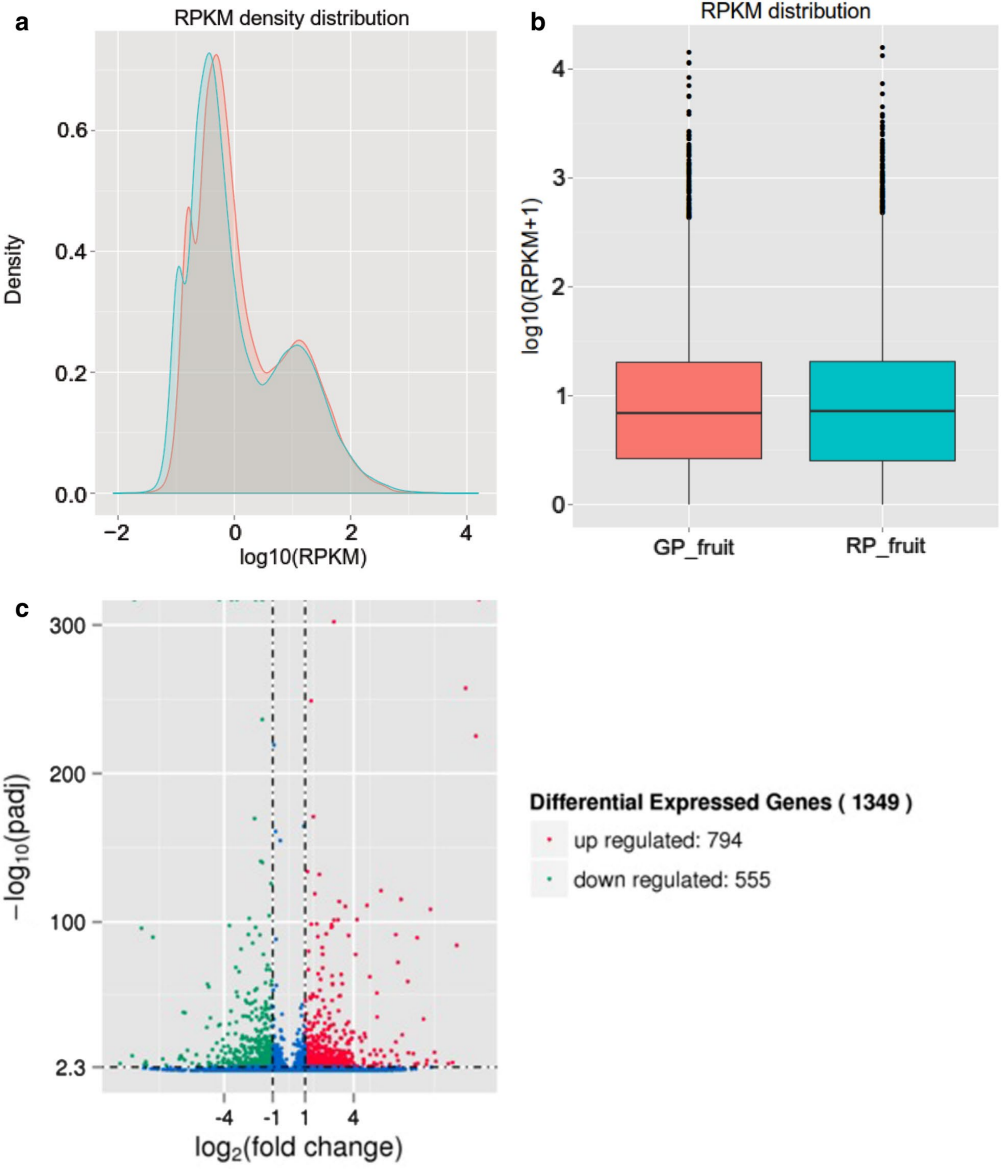
Distributions of gene expression value and volcano plots for the DEGs. **a** RPKM density distributions of all *D. longan* unigenes in the *green*- and *red*-peel fruit group. The *x-axis* denote log_10_ (RPKM) value, and the *y-axis* denote the density of corresponding log_10_ (RPKM) value. The *red curve* denote GP fruit (GP_fruit) group, and the *green* denote RP fruit (RP_fruit) group. **b** *Box plot* of the log RPKM expression values in both groups. **c** The *x-axis* describes the fold change in expression levels between two groups. The *y-axis* shows the statistical significance expressed as −log_10_ (adjusted p value) from the comparison. Genes with log_2_ (fold change) > 1 and with −log_10_ (adjusted p value) >2.3, which is the equivalent of FDR adjusted p value <0.005, were defined as differentially expressed genes (DEGs). The *red* points denote up regulated DEGs and the *green* points denote down regulated DEGs

Between the RP and GP fruit groups, via comparing the two distributions of expression value of all corresponding unigenes, it could be intuitively concluded that highly similarity was existed in the two distributions (Fig. 6a). Moreover, as shown by the box-plot distribution of the log RPKM values in Fig. 6b, the median and the quartile values between the two groups were almost identical. The RPKM values of most unigenes (40.99 and 46.05 % in RP and GP fruit groups respectively) were in the range from 0.3 (its log value is −0.52) to 3.6 (0.56). The RPKM values of some unigenes (3.69 and 3.36 % in RP and GP fruit groups, respectively) were higher than 60 (its log value is 1.78). However, on the whole, between two distributions of log_10_(RPKM) expression value, some differences indicated that expression level in GP fruit group is slightly higher than values in RP fruit group (Fig. 6a).

To identify the genes that were differentially expressed between two groups, the significantly threshold of adjusted p value (<0.005) under Storey’s false discovery rate (FDR) control (also named q value) and the absolute value of log_2_ value of fold change (>1) were used to strive for reducing false DEGs.

As shown in Fig. 6c, the comparison of RP and GP revealed that 794 genes were significantly up regulated and 555 genes were down regulated. The number of up regulated genes was more than down regulated genes. In these 1349 DEGs, 1285 genes were annotated at least in one database.

To identify candidate genes associated with coloration of longan peel and obtain more insight into its molecular regulation, genome-wide gene expression profiling was used to compare “RP” and “GP” longan. In this study, we generated a total of 24,044 unigenes from the two samples, and using this dataset, 1349 significantly differentially expressed genes were identified, among them 32 genes related with peel color of longan were identified. Of these, 17 genes were significantly up-regulated and 15 genes were down-regulated (Table 2).

**Table 2:** Significantly differentially expressed genes between RP and GP longan

Gene_id	FDR	Gene_name_1	log2FC(RP_vs_GP)
comp72873_c0	7.99E−03	*CYP75A1*	−7.3142
comp12349_c0	1.08E−39	*DFR*	−6.4108
comp20174_c0	3.12E−03	*CYP75A6*	−3.6151
comp31601_c0	1.15E−08	*CYP75A1*	−3.4039
comp25528_c0	3.21E−18	*RhGT1*	−2.9167
comp23727_c0	5.76E−04	*5MAT1*	−2.1086
comp28138_c0	3.04E−13	*SUC1*	−1.9628
comp33644_c1	1.28E−17	*CYP75A7*	−1.8656
comp33644_c0	1.11E−15	*CYP75A1*	−1.8615
comp36598_c0	2.06E−39	*CYP75A6*	−1.6417
comp28010_c0	3.81E−06	*CYP75A6*	−1.6095
comp12187_c0	2.50E−04	*GT5*	−1.5025
comp26265_c0	5.23E−05	protein: Naringenin, 2-oxoglutarate 3-dioxygenase (*F3H*) query_name: FL3H_MALDO	−1.4831
comp34697_c0	6.17E−03	protein:Anthocyanin 3′-O-beta-glucosyltransferase (*3′GT*) query_name: ANGT_GENTR	−1.3784
comp32613_c0	9.62E−29	*CYP75A2*	−1.3476
comp29846_c0	3.94E−04	*CYP75A7*	1.1538
comp34207_c0	2.55E−09	*CYP75A3*	1.2766
comp29890_c0	1.81E−12	*CHI1*	1.4885
comp38510_c0	1.88E−05	*CURL3*	1.6442
comp13425_c0	1.43E−02	*ANS*	1.6521
comp32817_c0	4.50E−03	*ANS*	1.7905
comp23348_c0	2.34E−04	*ANS*	1.8615
comp32978_c0	1.59E−83	*TBA*	2.0535
comp30021_c0	1.72E−03	*5MAT1*	2.3048
comp14493_c0	1.94E−06	*LAX3*	2.3793
comp12768_c0	3.95E−99	*UGT84A2*	2.6419
comp41364_c0	9.20E−04	*RT*	2.6585
comp33129_c0	8.11E−06	*C2*	2.7134
comp34926_c0	4.98E−102	*CYP75A2*	2.7751
comp12129_c0	8.67E−05	*FGT*	2.8528
comp12185_c0	6.35E−04	*C1*	2.9949
comp13215_c0	2.05E−14	*C1*	5.2789

**Fig. 7:**
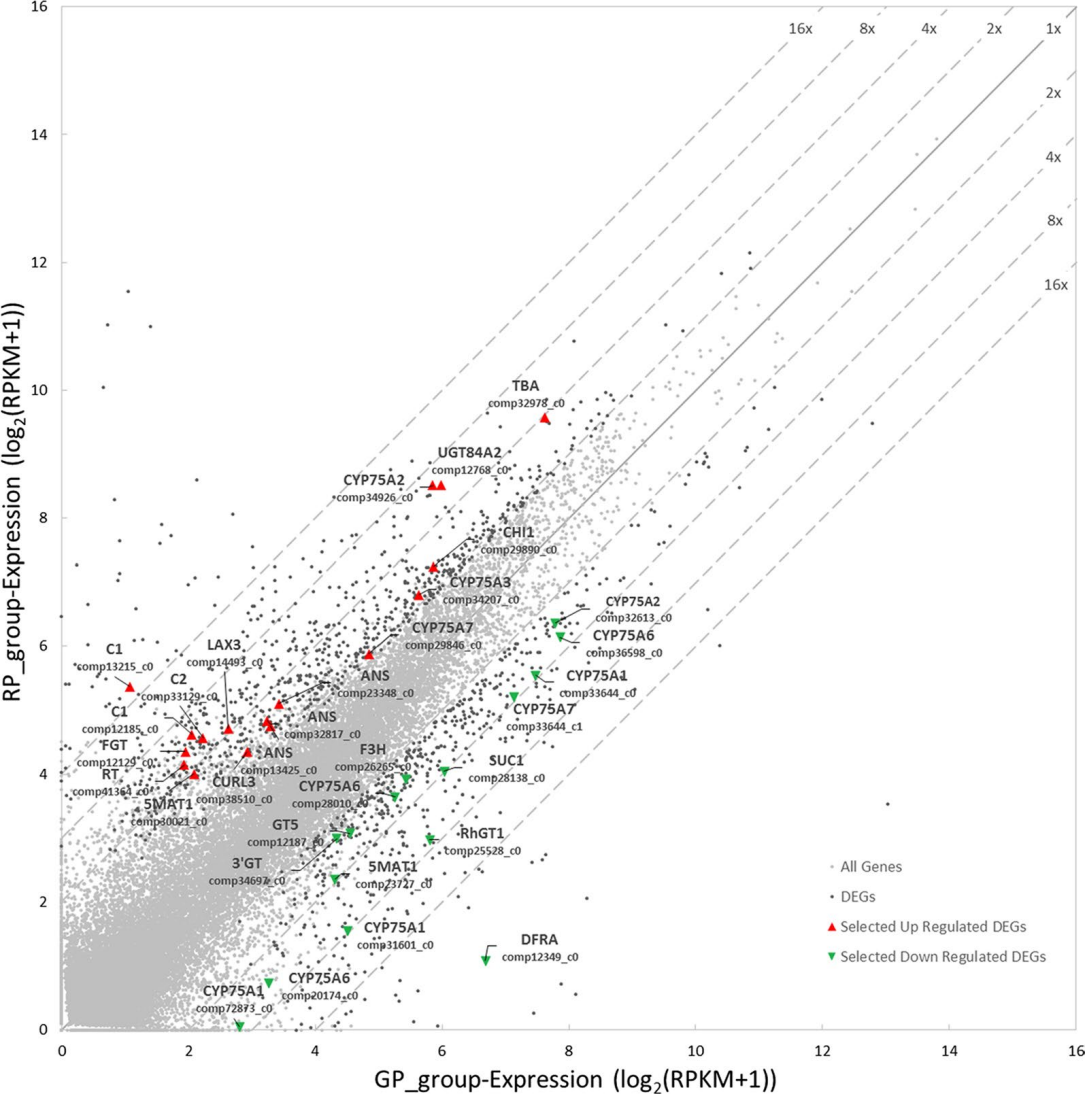
*Scatterplot* of transcriptome gene expression of longan with RP and GP

As well, RNA sequencing was used to examine differentially expressed genes between “RP” and “GP” longan, with the aim of identifying genes involved in regulating peel color. Our results suggest that differentially expressed genes, Dihydroflavonol-4-reductase (*DFR*), Flavonoid 3′,5′-hydroxylase1 (*CYP75A1*), Anthocyanin regulatory C1 protein (*C1*), whose expression abundances reached to 16-fold or greater changes in red peel compared with green peel (Fig. 7), may be important genes for regulating peel color in “RP” and “GP” longan, and they are all related with biosynthesis of anthocyanidins.

### Detection of variants in transcriptome

After aligning clean reads to transcriptome reference sequences, we used samtools and picard-tools to sort mapping results and remove multiple mapped reads. And then the GATK was implemented to detect the single nucleotide polymorphisms (SNPs) and short insertions/deletions (InDels). These raw variants were removed by the following conditions: (1) the quality of variant calling <30; (2) the distance of most neighbored variants <5.

**Fig. 8:**
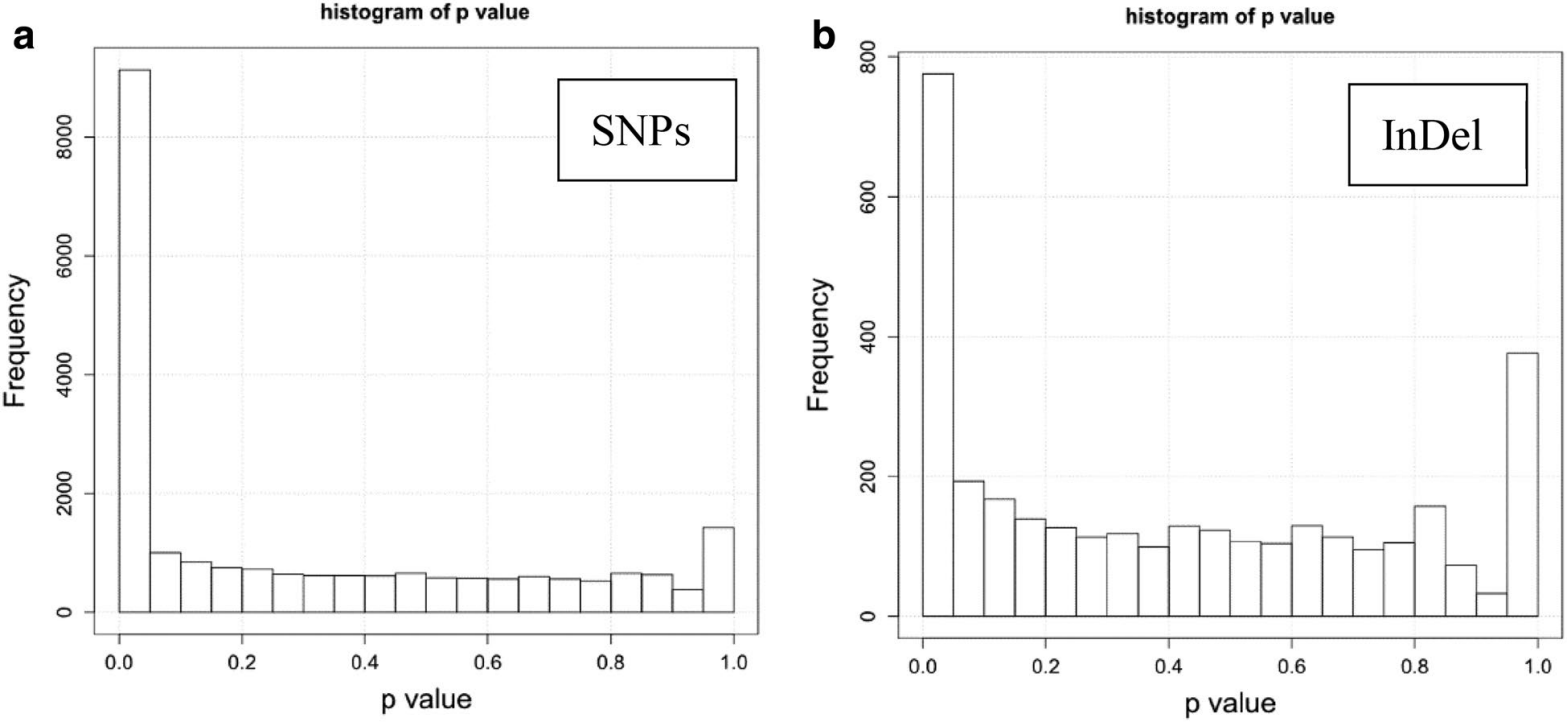
Histogram of p value calculated by Fisher’s exact test. The *left histogram* is SNPs (**a**) and the *right* is InDels (**b**)

As a result, 102,799 SNPs and 10,698 InDels were identified through the above method. Among these SNPs, 70,539 SNPs, were found in GP fruit group and 75,781 were found in RP fruit group. We also took advantage of information of predicted CDS to annotate these SNPs. Firstly, these SNPs could be divided into two categories: coding SNPs and non-coding SNPs. In the GP fruit group, there were 16,148 (22.89 %) coding SNPs and 54,391 (77.11 %) non-coding SNPs. And in the RP fruit group, there were 17,313 (22.85 %) coding SNPs and 58,468 (77.15 %) non-coding SNPs. Secondly, the coding SNPs could be subsequently annotated as synonymous or non-synonymous variant. Non-synonymous variants in our result were rare and meaningful. We only found 73 (0.10 %) non-synonymous SNPs in the GP fruit group and 67 (0.09 %) non-synonymous SNPs in the RP fruit group (Fig. 8).

To calculate the p value of independence in the 2 × 2 table (the two rows is the two groups and the two columns is the two alleles), Fisher’s exact test was applied for each variant. Before performing Fisher’s exact test, to guarantee that the sample size for statistical analysis is sufficient, an empirical value of lowest coverage depth at each variant site was set to 25 for both two groups. And finally, 22,116 SNPs and 3282 short InDels meet the above requirement. As shown in the Fig. 8, we found 9131 (41.29 %) SNPs and 776 (23.64 %) InDels had significant p value (p < 0.05).

## Discussion

Coloration of exocarp is mainly associated with accumulation type, quantity and distribution of anthocyanin, whose synthesis mechanism is not only about basic research of the fruit industry, it is also about consumer orientation. So far, there is no completely parallel key factor acting on anthocyanin biosynthesis pathway among different species, even the same. All the different/same species have their own specific expressive properties, especially the fruit types of aril, such as longan, litchi, representing significant differences of anthocyanins biosynthesis pathway from other fruits. Therefore, making them clear whether structural genes express in peel of special longan germplasm resource, which genes are the key ones for coloration of longan peel, and how the synthetic genes co-expressed is of great significance for elucidating the molecular basis of coloration of longan peel.

Over the past decade, the development of transcriptomic and genomic technologies has contributed to a better understanding of coloration of fruit peel at the molecular level. However, most of our knowledge about peel coloration has arisen from studying coloration regulatory genes in many other fruits except longan. A lack of genetic information hinders our research, but at the same time, it also makes our study more essential.

GO annotation, whose functional interpretations for plants are primarily based on the *Arabidopsisthaliana* genome, is able to provide a description of gene products in terms of their associated molecular functions, cellular components, and biological processes (Berardini et al. 2004). The GO annotations of the unique genes were most frequently related to biological processes (29,618 unigenes), followed by molecular function (12,489 unigenes) and cellular components (8036 unigenes). For each unigene, the specifically annotated GO terms provide a broad overview of the groups of genes cataloged in the transcriptome, and the GO annotations provided valuable clues to investigate the biological processes, molecular functions, and cellular structures of the *Dimocarpus* Lour. transcriptome.

The KEGG classification system, integrating current knowledge on molecular interaction networks, provides an alternative functional annotation of genes according to their associated biochemical pathways (Kanehisa et al. 2004). Metabolic pathways were well represented among longan peel unique sequences, most of which were associated with carbohydrate metabolism, translation, folding, sorting and degradation. Overview, amino acid metabolism, environmental adaptation, lipid metabolism, energy metabolism and biosynthesis of secondary metabolites were included in the top 19 pathways. Flavonoid biosynthesis is an integral part of secondary metabolism, and the transcripts encoding some enzymes involved in the flavonoid biosynthesis pathway, such as *DFR*, *F3H*, and *ANS* were present in our Illumina sequences dataset (Table 2); therefore, flavonoid biosynthesis pathway should be considered within the context of peel coloration of longan.

With the aim of identifying genes involved in regulating peel color, RNA sequencing was used to examine differentially expressed genes between “RP” and “GP” longan in this study, and we speculated the peel color of longan may be involved in the flavonoid metabolism pathway, especially the anthocyanin biosynthesis.

*DFR*, *F3H* and *ANS* are parts of important steps in the flavonoid biosynthetic pathway of anthocyanins, respectively, they convert dihydroflavonol into leucoanthocyanidin, naringenin into dihydrokaempferol, leucoanthocyanidin into anthocyanin in the anthocyanin biosynthetic pathway (Ahmed et al. 2014; Holton and Cornish 1995; Lin et al. 2013). Especially, *DFR*, whose expression abundances reached to 16-fold change in red peel compared with green peel, presented the most significantly different expression among *DFR*, *F3H* and *ANS*. DFRA is the key enzyme for the biosynthesis of anthocyanins in the skins of peach and nectarine fruit (Zhou 2009). In addition, regulating the expression of *DFR* can change the flower color in Japanese parsley, tobacco and petunia (Yamaguchi et al. 2004). The substrate specificity of the *DFR* often determines which anthocyanidins a plant accumulates (Magnus 1999). In purple kale (*Brassica oleracea var. acephala f. tricolor*), the expression of anthocyanin biosynthetic gene *DFR* is enhanced in response to low temperature treatment (Zhang et al. 2012). In conclusion, the *DFR* gene has different expression pathways in different plant species; therefore, the function of *DFR* in the anthocyanin biosynthesis process remains to be further investigated.

The *CYP75A1* gene, belongs to the *CYP75A* subfamily, which is able to catalyze the 3′5′-hydroxylation of naringenin and eriodictyol to form 5,7,3′,4′,5′-pentahydroxy flavanone and 3′,5′-hydroxylation of dihydrokaempferol and dihydroquercetin to form dihydromyricetin. Flavonoid 3′,5′-hydroxylase**(***F3′5′H*) is necessary for biosynthesis of the anthocyanins that confer a violet or blue color to most plants (Tanaka et al. 2008). In berry, *F3′5′H* gene expression has a functional impact on anthocyanin biosynthesis that persists during fruit ripening, and among red grape varieties, expansion and sub-functionalization of *F3′5′H*s have increased the complexity and diversification of the fruit color phenotype (Falginella et al. 2010). As well, in the grapevine lineage, higher levels of *F3′5′H*s transcription in dark blue cultivars than light red cultivars, even in green-peel cultivars, *F3′5′H* transcripts are completely absent (Mattivi et al. 2006; Pomar et al. 2005). In this study, expression abundance of *CYP75A1* was very low in RP longan, further study should focus on its biologically significance combined with related background knowledge.

*C1* is a regulatory gene of the anthocyanin pathway, which regulates the expression of at least three structural genes: chalcone synthase, dihydroflavonol reductase and flavonol O_3_ glucosyltransferase. In the past decades, research on *C1* gene mainly focus on maize (*Zea mays*) (Avila et al. 1993; Franken et al. 1994; Köhler et al. 1995; Petroni et al. 2000; Piazza et al. 2002; Scheffler et al. 1994). In maize, *C1* is required for anthocyanin synthesis only in seed tissues, and different light treatments affected the expression level, white, red, and blue light were effective in stimulating anthocyanin accumulation and expression of the *MYB*-related gene (Piazza et al. 2002). The accumulation of *C1* transcript is under both developmental and light control (Franken et al. 1994), and in recent years, many studies indicated that *MYB* played an important role in regulating peel color (Espley et al. 2007; Feng et al. 2013; Niu et al. 2010; Rahim et al. 2014; Schwinn et al. 2006; Sun et al. 2013; Yang et al. 2015). In our study, *C1* gene was detected as a significantly DEG, we presumed the light regulation of transcription factors may control anthocyanin biosynthesis in longan peel.

We have been aware that genetic evidences are needed to support these hypothesis on function gene *DFR*, *F3H*, *ANS*, *CYP75A1* and *C1*, which were speculated to play an important role in formation of peel color in longan. The expression abundance of *DFR*, *F3H*, *ANS*, *CYP75A*, and *C1* and the accumulation of anthocyanin in RP and GP are worthy of further investigation, as well as their SNPs. With the aim of revealing the molecular mechanism of regulating peel color deeply in woody plants, the quantitative real-time PCR (qPCR) and high-performance liquid chromatography (HPLC) analysis should be respectively applied to verifying the expression levels of these genes and total anthocyanin concentration (Additional file 1).

## Conclusions

 To summarize, this work is the first report of gene-expression profiling in longan skin conducted by Illumina next-generation sequencing technology. We identified the genes encoding key enzymes involved in flavonoid biosynthesis pathways, which are most likely to play an important role in peel color of longan. Besides, the accumulation of flavonoids and the expression levels of genes associated with their biosynthesis and metabolism in longan peel are worthy of further investigation, which could help provide insights into the molecular mechanisms of regulating peel color in woody plants (Additional file 2).

## Additional files

10.1186/s40064-016-2743-y RNAseq Raw files for GP fruit and RP fruit.
10.1186/s40064-016-2743-y Authors' original files for Gene Functional Annotatiion, GO Classification, KOG Classification, KEGG Classification, and SNP Calling.

## Abbreviations

NR: NCBI non-redundant protein sequence
NT: NCBI non-redundant nucleotide sequences
KOG: euKaryotic Ortholog Groups database
CDS: Coding Sequence
Swiss-Prot: manually annotated and reviewed protein sequence database
KEGG: Kyoto Encyclopedia of Genes and Genomes
KO: KEGG ortholog
GO: gene ontology
PFAM: protein family
DEGs: differentially expressed genes
SNP: single nucleotide polymorphisms
RP: red peel
GP: green peel
*DFR*: dihydroflavonol-4-reductase
*F3H*: chalcone synthase flavanone 3-hydroxylase
ANS: anthocyanidin synthase
*CYP75A1*: flavonoid 3′,5′-hydroxylase 1
*C1*: anthocyanin regulatory C1 protein
